# If You’ve Got It, Flaunt It: Humans Flaunt Attractive Partners to Enhance Their Status and Desirability

**DOI:** 10.1371/journal.pone.0072000

**Published:** 2013-08-15

**Authors:** Benjamin M. Winegard, Bo Winegard, David C. Geary

**Affiliations:** 1 Department of Psychological Sciences, The University of Missouri, Columbia, Missouri, United States of America; 2 Department of Psychology, Florida State University, Tallahassee, Florida, United States of America; University of Nottingham Malaysia Campus, Malaysia

## Abstract

Mating decisions are influenced by conspecifics’ mate choices in many species including humans. Recent research has shown that women are more attracted to men with attractive putative partners than those with less attractive partners. We integrate these findings with traditional accounts of social signaling and test five hypotheses derived from it. In our study, 64 men and 75 women were paired with attractive and unattractive opposite-sex putative partners and asked whether they would prefer to give surveys to peers or to older adults. Consistent with predictions, both men and women wanted to show off (flaunt) attractive partners by administering surveys to peers and both men and women wanted to hide (conceal) unattractive partners from peers by administering surveys to older adults. These decisions were mediated by how participants expected others to evaluate their status and desirability when they administered the surveys, consistent with partners serving a social signaling function in humans.

## Introduction

In an amusing and insightful scene from the movie Legally Blonde, a snobby woman emphatically rejects a hapless man (“women like me don’t date losers like you”). Walking by, Elle Woods (played by Reese Witherspoon) hears the exchange, confronts the man in front of the woman, and feigns that he had broken her heart. The other woman, surprised, suddenly changes her attitude, asking the once unappealing man, “so when did you want to go out?” This is a humorous example of nonindependent mate choice or mate choice influenced by the decisions of same-sex peers [1]. Nonindependent mate choice has been found in many species including humans [2], [3], but is not well integrated with existing evolutionary theories of human mating behavior. We believe that signaling theory, a modern theoretical development that analyzes the logic of animal communication, enables this integration. Specifically, we propose that mates can function as honest signals of social status and mate value and thus mates will be flaunted or concealed, depending upon their relative signaling value. Below, we present our proposal and test five hypotheses derived from it in a study in which young women and men paired with attractive and unattractive opposite-sex partners chose whether to administer a face-to-face survey to their peers (flaunting) or to older adults (concealing).

### Nonindependent Mate Choice

In the early 1990’s, biologists began to document cases of nonindependent mate choice (also termed “mate choice copying”) in several species of animal, including guppies and sage grouse [1]. In these species, females preferentially select males that are seen in proximity with other females (and thus have likely mated with those females). Two reasons are generally offered to explain this phenomenon: cost avoidance and improved discrimination. The first posits that females use public mating information to select a mate without bearing the costs of active mate search and choice. The second posits that females use public information, augmenting their own assessments with the assessments of other females, to improve the quality of their mate selection [4].

Vakirtzis and Roberts [4], [5], [6] added important theoretical refinements to this literature. They noted that mate copying, or simply imitating the decision of any female in the population, would be unlikely to arise in a relatively monogamous species; rather, what would arise is a form of nonindependent mate choice that they termed “mate quality bias.” Mate quality bias is a subset of nonindependent mate choice where females assess the quality of a male’s mate and use this information in making their own mate choice decisions. In other words, assortative mating results in a reliable correlation between the quality of a male and the quality of his partner, and this correlation can be used to enhance mate choice discrimination. Because humans are relatively monogamous, Vakirtzis and Roberts proposed they should exhibit mate quality bias, not mate choice copying, i.e., women will be influenced by the quality of a man’s partner not simply the fact that he has a partner [4], [5].

Modern studies, using various methods, indicate that a man and/or woman’s putative partner affects observers perceptions of his or her traits [9], [10], [11]. Sigall and Landy [7], for example, found that both men and women rated a man who was associated with an attractive, opposite sex confederate more favorably than when he was not associated with an attractive confederate. Observers rated a man as least favorable when he was associated with an unattractive confederate. Strane and Watts [8] found that women who were paired with attractive men in photographs were rated more favorably than women who were paired with unattractive men. Waynforth [12] and Vakirzis and Roberts [13] explicitly demonstrated that the variable of importance in these cases is the relative attractiveness of the putative partner. That is, observers rate a male more favorably if he is with or desired by an attractive putative partner than if he is with or desired an unattractive putative partner, supporting Vakirtzis and Roberts’ contention that humans should exhibit mate quality bias, not indiscriminate mate choice copying.

Although mate quality bias better explains the existing data, it fails to integrate such effects into a broader, more parsimonious theory. We believe that applying signaling theory to these data has the potential to provide this integration.

### Signaling and Social Information

Signaling theory has been successfully used to understand the evolution and expression of costly physical and behavioral traits, such as the elaborate plumages of peacocks, the beautifully wrought bowers of bowerbirds, and the sprightly stotting of gazelles [14], [15]. The basic tenets of signaling theory, as applied in evolutionary biology, are straightforward. Trait quality varies among individuals of all species. These traits are not always manifested or easily perceivable (e.g., dominance, intelligence, immunocompetence) but can, in principle, be reliably signaled by other traits. Thus perceivers benefit by attending and reacting to the signals. However, signalers and perceivers often have competing interests. The signalers can potentially exploit the perceivers by enhancing their signals without changing underlying traits; therefore, perceivers must remain vigilant against deceptive signals. A solution to this potential arms race is the development of signals that are difficult to fake and therefore reliable indicators of the underlying traits [16]. This, in turn, explains why many animal signals, especially ones sent between animals with competing interests, are extravagant: such signals are costly and thus cheating is difficult. However, there are other mechanisms and constraints that help ensure honesty, and it has become clear in recent years that Zahavi’s contention that signals require exorbitant costs is incorrect [17], [18]. Cost, in other words, is only one mechanism that deters deceptive signals.

Although humans have developed the most abstract and sophisticated signaling space among extant species, theorists have argued that the basic principles are the same. These theorists, influenced by Veblen’s [19] early work on conspicuous consumption, have used those principles to illuminate otherwise puzzling aspects of human social behavior. Miller [20], for example, argues that one reason the human brain is so unique is because it is a signal sending organ, and that many of the cultural products that it produces–sublime sculptures, gripping novels, eloquent poems–are signals of the producer’s underlying mate quality. Other scholars, applying signaling theory to archaeological data from the Great Basin, have argued that men shifted to large game hunting because they were competing to send reliable signals of their underlying qualities, such as strength, intelligence, and generosity [21]. More recently, Miller [22] extended costly signaling logic to consumerism, arguing that consumer products are used to advertise underlying traits. Although there is debate over the precise signaling functions of consumer products, most researchers agree that prestige goods such as Porsches, Rolexes, or rare and aged scotches, are reliable signals of status and wealth and perhaps other traits [22], [23] because obtaining them is difficult for lower status individuals. Consistent with the proposal that they function as honest signals of wealth, status, and other desirable traits, once a prestige good is easily copied or accessible, its value declines and it is replaced by another prestige good or an elaboration of the old good [24]. Whether such prestige goods function like biological signals is controversial, and researchers are skeptical that biological signals and prestige good signals adhere to the same underlying logic [25]. Cronk [26] noted that it is times more useful to use the term “hard-to-fake” signal than “costly” signal to avoid the possible pitfalls of misleading terminology. What is important for the purposes of this article is that mates function as an honest signal of a person’s underlying traits.

In what follows, we build upon these general principles (with noted caveats) and extend them to nonindependent mate choice by asserting that mates can function, in part, as social signals; that is, mates, like other luxury goods, convey social information about their partners’ traits or qualities that are not readily observable.

### Social Signaling and Nonindependent Mate Choice

Humans are motivated to display signs of social and cultural status [22], [27], [28], not only to obtain desirable mates but also to gain social influence and through this access to other types of resources. One fundamental, but often overlooked, signal of a person’s status is his or her mate, because highly desirable mates are scarce, coveted, difficult to obtain, and require more investment than less desirable mates [29]; or, put more generally, desirable mates are honest indicators of their partners’ positive traits, including status, wealth, and, arguably, genetic quality. If so, the principles of signaling theory should generalize to mating, and humans should either flaunt or conceal their partners depending upon the partners’ relative mate value–a prediction Vakirtzis and Roberts [5] derived from the mate quality bias perspective. By “flaunt” we mean actively display, show off, or boast about a mate and by “conceal” we mean actively hide or remain silent about a mate. Although this signaling system is predicted to be reliable, there is a potential for deception. Perceivers should be motivated to determine the nature of an ambiguous relationship, and thus detect “cheats,” using social information such as gossip, and honest displayers should desire to make their signals unambiguous by using socially accepted advertisements of partnership (e.g., holding hands, kissing in public, exchanging jewelry, facebook status) [30]. Perceivers evaluating the relationship should also attend to disparities in affection between partners. Other things equal, individuals of high mate value or social status are predicted to expend less effort on winning and sustaining the affections of an attractive mate; therefore, perceivers should impute more status and mate value to flaunters who appear less affectionate than their partners, a fact that is often parlayed to enhance (or make explicit) the status and mate value of fictional characters like James Bond or Scarlett O’hara.

The extension of signaling theory to mates straightforwardly predicts that the quality of a person’s mate will affect the perceptions and decisions of other potential mates (nonindependent mate choice), consistent with existing data [3], [7]. The extension is also consistent with a number of hypotheses that are not readily deducible from existing mate quality bias literature. Two of the most important and easily testable predictions are that both men *and* women: 1) will flaunt (tested here) [31], and 2) flaunt to same sex as well as opposite sex peers (a prediction we have recently confirmed, unpublished data); flaunting to same sex individuals is predicted to enhance social influence. On the basis of signaling theory, both sexes possess traits that contribute to their social influence and mate value but are not easily perceivable [20]; therefore, both men and women should publicly advertise hard-to-fake perceptible signals (including mates) that broadcast possession of these hidden traits. As noted, previous research shows that both males and females who are paired with attractive partners are more positively evaluated by both same and opposite sex observers [7], [8]. Although the proposed extension of signaling theory, consistent with other research [32], predicts that increased (or primed) mating motives may augment an individual’s propensity to flaunt, it also predicts that an individual will flaunt or conceal regardless of his or her relationship status. This for two reasons: First, humans seldom remain in a single romantic relationship for life and, therefore, they should remain concerned with their status and mate value even when in a secure relationship. And, second, the status that accrues to humans who flaunt valued mates can be used to increase fitness through enhanced social influence generally, independently of securing other (presumably higher valued) mates. The most direct way is through passing acquired resources and status to offspring [33].

### The Current Study

We derived and tested five hypotheses from our extension of signaling theory. First, men will flaunt and conceal. Second, women will flaunt and conceal. Third, men will flaunt and conceal a partner’s attractiveness more than women. The latter prediction is based on the design of our study. In it, we manipulated only the attractiveness of a participant’s putative partner. Men’s mate choices are more strongly influenced by physical attractiveness than women’s choices [28] and thus physically attractive mates have higher signal value for males than females, therefore this manipulation should result in a stronger motivation to flaunt or conceal the partner in men than women. Fourth, flaunting and concealing will not be moderated by the relationship status of the participants. Because this is a null prediction, it is not strong and the results should be interpreted with caution. Fifth, the relationship between partner attractiveness and flaunting/concealing will be mediated by expected impact on the flaunters’ status and desirability. This final prediction stems from our proposal that flaunters signal to increase or broadcast their status and mate value; we used “desirability” as a substitute for “mate value,” which is unclear to participants. Although we do not believe that flaunters or concealers need to be conscious of why they are flaunting or concealing, we do believe that, as with the display of other status signals, they are conscious of the consequences [34].

## Materials and Methods

### Ethics Statement

All participants completed written informed consent forms before participating in the study. Consent forms were collected and stored in a locked cabinet. After completing the study, participants were first orally debriefed and then given a written debriefing form documenting the nature of our experiment. This study and the consent and debriefing procedure were reviewed and approved by the University of Missouri Institutional Review Board.

### Participants

Undergraduates at the University of Missouri participated for partial course credit. The sample consisted of 64 men (mean age = 20. 27 SD = 4.86) and 75 women (mean age = 19.10, SD = 1.87). Of these, 21 men and 28 women reported being in a relationship [Men mean relationship satisfaction = 4.23, SD = 1.26; women mean = 4.28, SD = 1.18; see Supplementary Information S1).

### Design and Procedure

Participants were given pamphlets (see Pamphlet 1 in Supplementary Information S3 and Pamphlet 2 in Supplementary Information S4) dealing with higher education surveys. They were told that they would collect survey data about attitudes toward higher education and educational funding from one of two areas on campus. Participants were told we were interested in response rates and how the characteristics of the surveyors affected respondent’s answers. Participants in the experimental condition were told they would be collecting their data with a partner and were to act as if they and their assigned partner were in a happy romantic relationship (participants were told this had been shown to affect response rates). After completing basic demographic questions, participants filled out the Ten Item Personality Measure [35]. This measure has low reliability and therefore was included as a filler (rather than being used in subsequent analyses) so that participants would not become suspicious about the veracity of our cover story.

Participants were then told that pictures of their partners had been collected so they could be identified when they met to collect survey data. To ensure realism, participants were told that participants who did not want their pictures shown were removed from the study and given credit for participating in a separate study. Participants then turned the page of the pamphlet and looked at the approximately three by three inch black and white photographs of their putative partners; each participant had only one randomly assigned putative partner. The photographs were taken from a pre-rated (raters were eight men and eight women) set of 247 photographs (117 men and 130 women); in this larger set, photographs that did not appear natural (e.g. were idiosyncratically lit) were removed. From that pre-rated set, we chose three women and three men from the top decile of attractiveness and three women and three men from the bottom decile, ensuring that the attractive putative partners were very attractive and that the unattractive putative partners were very unattractive (see Supplementary Information S2).

Underneath their picture, each putative partner had a name and a schedule of open times during which they could meet in the following two weeks to conduct the surveys. Participants were asked to mark the number of hours they were available to meet. Next, participants read about two locations that they could choose to conduct the surveys. One location was described as full of undergraduate students and one was described as full of administrators (see pamphlet S3). Participants were then asked to pick a location preference on a 1 (strongly prefer the administrative location) to 7 (strongly prefer the undergraduate location) Likert scale. Finally, participants filled out six questions on a four point scale about how they would feel as they collected the survey data and seven questions on a five point scale about how they thought they would be perceived as they collected the survey data (e.g. “Other people will view me as having status.”; 1 = strongly disagree and 5 = strongly agree). After participants completed the pamphlet, participants were told that the experiment was over and were orally debriefed (see debriefing in S1). The control group was run in a similar manner. They received pamphlets and were told that they would administer surveys in one of two locations (see pamphlet S4). They were then told that participants would give out the surveys with partners, but that they would be alone; therefore, their pamphlets did not have pictures of putative partners. They then filled out the same questions as the experimental groups, were told that the experiment was over, and were orally debriefed.

### Operationalization of Flaunting and Concealing

Because the undergraduate location consists of peers whose judgments are likely to be salient and relevant to the participants, it was considered the flaunting location, and the administrative location, because it consists of people outside of the participants’ relevant peer group, was considered the concealing location [36]. Flaunting is operationalized as a significant preference for the undergraduate location compared to the preference of the same-sex control group; and concealing is operationalized as a significant preference for the administrative location compared to the preference of the same-sex control group. Because we were also interested in sex differences in flaunting/concealing, we first conducted a 2 (sex) by 3 (partner attractiveness: attractive, unattractive, and control) ANOVA to test whether location preference differed across conditions and to test the sex by condition interaction. Planned comparisons using the Mean Square Residual of the ANOVA were then conducted to investigate each specific hypothesis separately for each sex (e.g., for the hypothesis “men will flaunt,” we compared men in the attractive condition to men in the control condition) and across each sex in the flaunting and concealing conditions (e.g., for the hypothesis “men will flaunt to a greater degree than women,” we compared men in the attractive condition to women in the attractive condition). Because these tests were explicitly based on a priori hypotheses, we did not control for multiple comparisons.

## Results

The 2 by 3 ANOVA with location preference (undergraduate to administrative) as the dependent variable confirmed the predicted main effect of partner attractiveness, F(2, 133) = 27.35, p<.0001, ηp^2^ = .292, and the sex by partner attractiveness interaction, F(2, 133) = 4.23, p = .02, ηp^2^ = .060.

**Figure 1:**
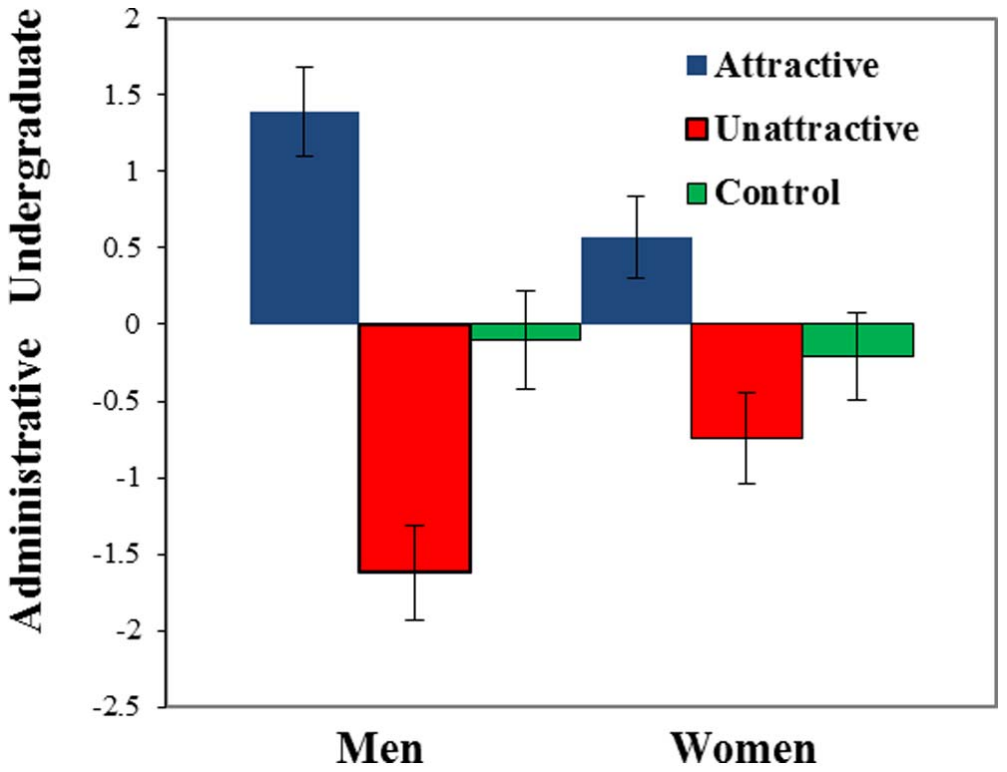
Flaunting and concealing as a function of sex and partner. Note. 0 represents no location preference and is considered neutral (4 on the 7-point location preference scale). We calculated preference for undergraduate or administrative location by subtracting the participants’ scores by 4 (no location preference). Bars represent standard errors.

**Figure 2:**
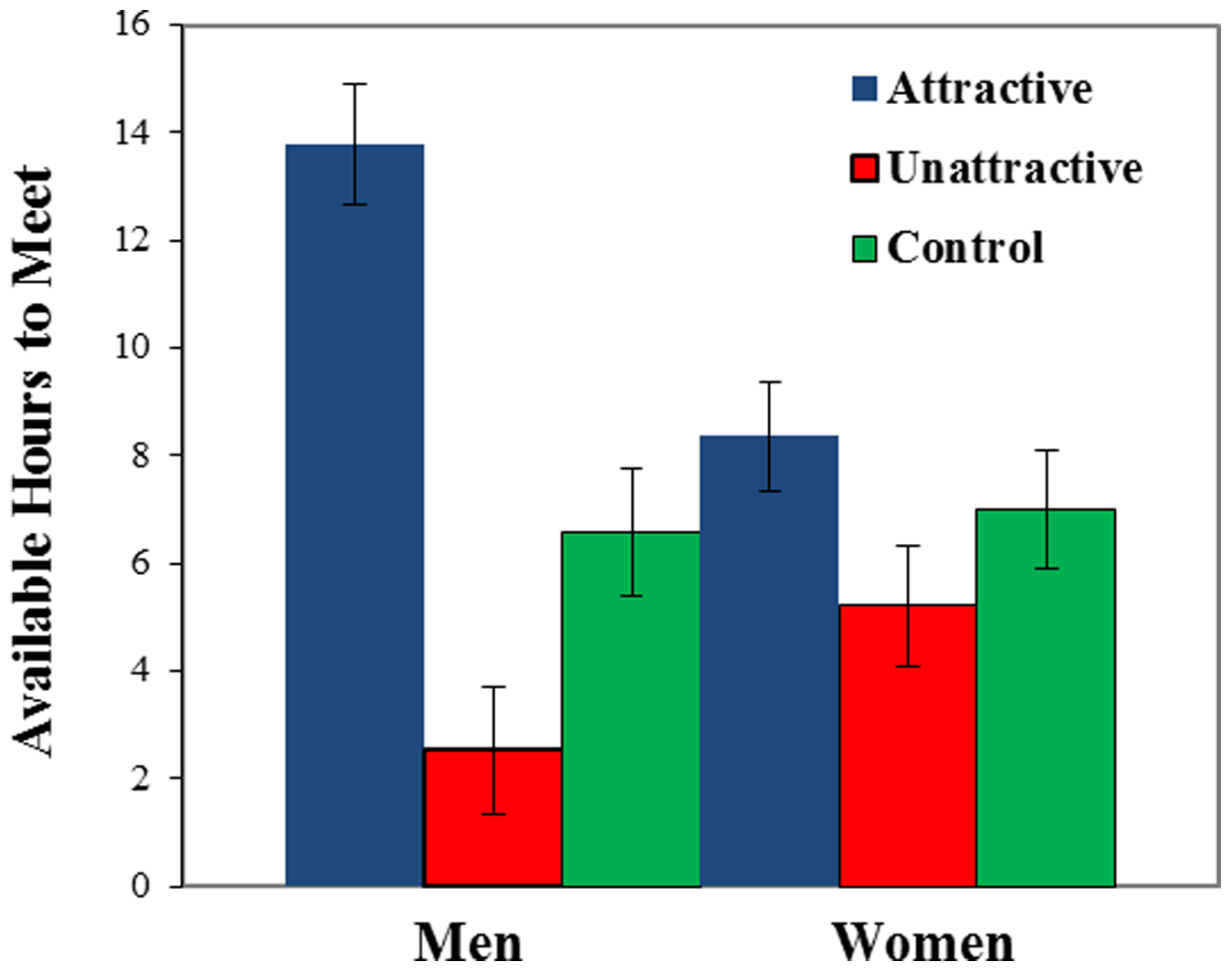
Hours available as a function of sex and partner. Note. Numbers represent the times participants reported being available to meet with their putative partner (see pamphlets in Supplementary Information S3 and S4). Bars represent standard errors.

The first hypothesis, that men would flaunt and conceal, was supported (see Figure 1, Table 1): in comparison to men in the control group, men flaunted attractive women (d = 1.13, p = .001) and concealed unattractive women (d = 1.14, p = .001). The second hypothesis, that women would flaunt and conceal was partially supported: in comparison to women in the control group women flaunted attractive men (d = .55, p = .05), but did not conceal unattractive men (d = .33, p = .20). The third hypothesis, that men would flaunt and conceal more than women, was also supported: men (M = 5.39, se = .30) flaunted more than women (M = 4.57, se = .27, p = .042, d = .58), and men (M = 2.38, se = .31) concealed more than women (M = 3.26, se = .30, p = .042, d = .65). Convergent support is provided by the finding that both men and women who were paired with attractive partners reported more hours available to meet with their partner in the following two weeks than those paired with unattractive partners (see Figure 2; see also S3 and S4). Participants’ reported hours available were also significantly correlated with how much they flaunted or concealed their partners (r = .43, p<.01).

**Table 1:** Summary Results for Flaunting and Concealing Hypotheses.

Hypothesis	Supported?	t-value	p	Cohen’s d
**Hypothesis 1**				
Men flaunt	Yes	3.44	.001	1.13
Men conceal	Yes	3.43	.001	1.15
**Hypothesis 2**				
Women flaunt	Yes	1.98	.050	0.55
Women conceal	No	1.28	.202	0.33
**Hypothesis 3**				
Menflaunt>women	Yes	2.06	.042	0.58
Menconceal>women	Yes	2.05	.042	0.65

Our first three hypotheses with specific predictions. Flaunting is defined as a significant preference for the undergraduate location compared to the mean of the control condition. Concealing is defined as a significant preference for the administrative location compared to the mean of the control condition.

A moderation analysis supported hypothesis four, showing that a participant’s flaunting/concealing was not moderated by his or her relationship status (in relationship, single) [F(2, 133) = .083, p = 0.77].We ran further exploratory moderation analyses on collected variables (see Additional Moderator Analyses in Supplementary Information S1) and found that religion significantly moderated location preference; specifically, participants who reported no religious affiliation were significantly more likely to flaunt than those who reported being religious [t(36) = 2.45, p = .02, d = 1.02; Figures S1 and S2 ].

**Figure 3:**
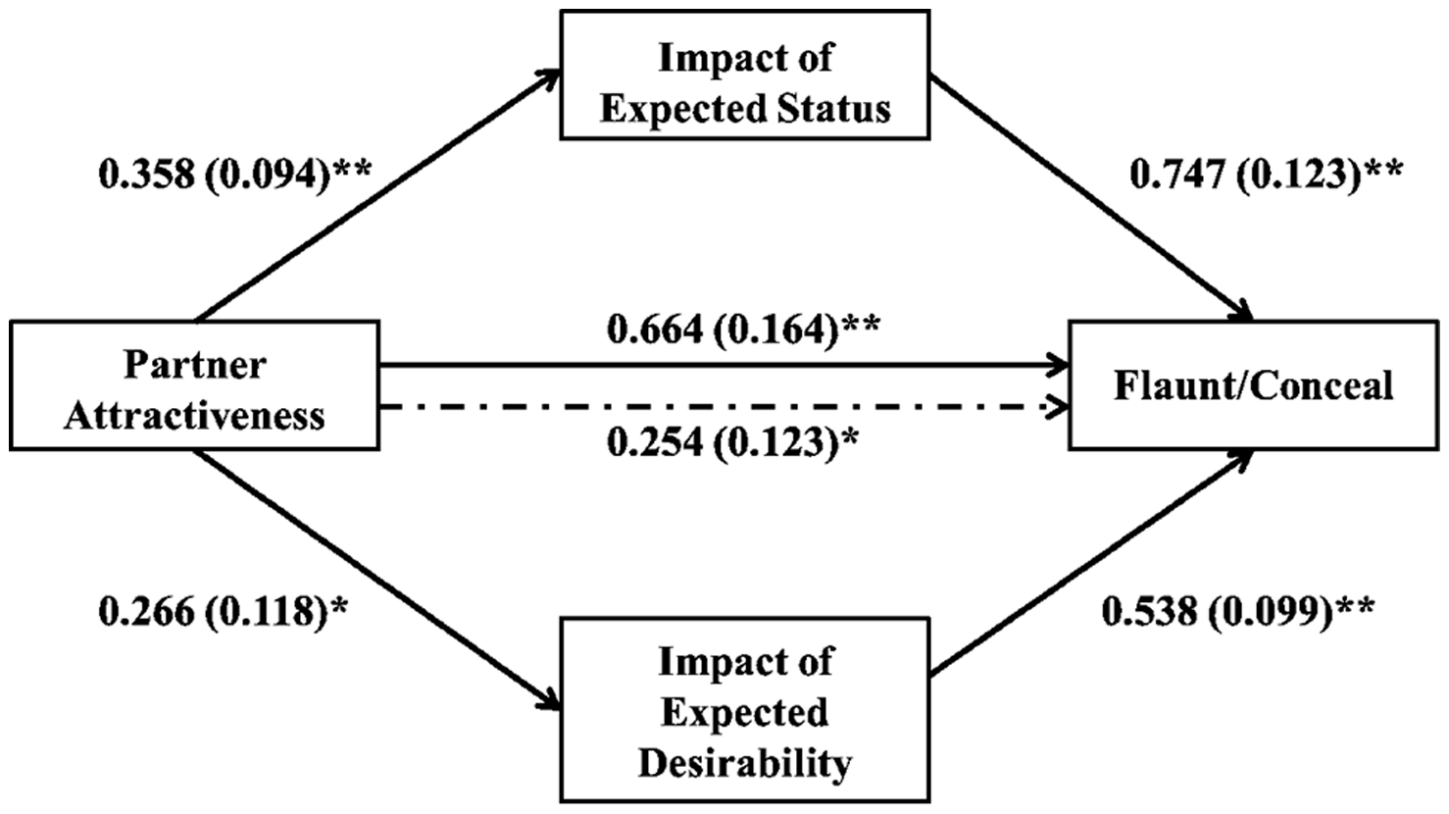
Mediation model showing the relation between partner status and flaunting/concealing as mediated by the expected impact on status and desirability. Note. Unstandardized coefficients illustrated with standard errors in parentheses. One asterisk indicates (p<.05); two asterisks indicate (p<.01). Solid line from IV to DV shows the total effect of the IV on the DV (c path); the dashed line shows the direct effect of the IV on the DV (c-prime path).

To test hypothesis five, that expected impact on status and desirability would mediate the relationship between partner attractiveness and flaunting/concealing, we used Preacher and Hayes’ [37] macro designed for SPSS to run a multiple mediation model. Confidence intervals were estimated with bootstrap analysis, a non-parametric method that is robust and does not assume multivariate normality. The results revealed that both the impact of expected perceived status and the impact of expected desirability partially mediated the relationship between partner attractiveness and flaunting/concealing. Specifically, the total effect (including indirect and direct effects) of partner attractiveness on flaunting/concealing was 0.664 (p<.01), whereas the direct effect (the effect after removing the indirect effect of the mediating variables) was 0.254, (p = .04; Figure 3 and Table 2). Mediation results were similar for both men and women and single participants and participants in a relationship (see additional mediation analyses in S1). Because there is a relationship between status and mate value (the constructs are not entirely unique), it is not surprising that there was a correlation between expected attributions of status and desirability (r = .53, p<.01); however, the multiple mediation macro takes this correlation into account and the indirect effects refer only to the unique capacity of each variable to mediate [37]. (see Table S1).

**Table 2:** Results Testing if Expected Status and Expected Desirability Mediated Tendency to Flaunt or Conceal.

Predictor	Indirect effect	95% CI	Z	p
Total sample (*N = *139)				
Expected status	0.267 (0.083)	[0.119, 0.451]	3.24	.001
Expecteddesirability	0.143 (0.068)	[0.039, 0.306]	2.10	.036
Overall	0.410 (0.122)	[0.195, 0.662]	3.37	<.001

Standard errors in parentheses. Confidence intervals estimated based on 1,000 bootstrap resamples. Model summary = R2 = 0.554, F(3, 135) = 55.81, p<.001.

## Discussion

To our knowledge, our results are the first to provide experimental evidence of mate flaunting, an effect that was found for both sexes. We also found that men, but not women, conceal unattractive putative mates, which is consistent with our hypothesis that men would conceal more than women; however, we also predicted that women would still conceal, if not as actively as men, but this prediction was not supported by our results. Also consistent with our prediction, relationship status did not moderate flaunting/concealing behaviors. Finally, as predicted, the expected impact of being seen with an attractive or unattractive putative partner on perceived status and desirability partially mediated the relationship between partner attractiveness and flaunting/concealing.

Although we did not predict that flaunting/concealing effects would be moderated by religious affiliation, we found that non-religious individuals flaunted to a greater degree than religious individuals. We believe that this result may provide convergent evidence for our proposal that humans flaunt to procure status and that such flaunting is similar to the flaunting of a luxury good. Recent research demonstrates that those who score higher than others in spirituality are less likely to endorse or participate in material based conspicuous consumption [38]. If people flaunt mates for the same reasons they flaunt for materialistic status, then our religiosity results are straightforwardly connected to these previous findings, although we did not predict them. Perhaps, as suggested by Weeden, Cohen, and Kenrick [39], religious (and, by extension, spiritual) concerns are, to some degree, driven by a particular reproductive strategy that is not directly linked to materialistic status concerns. Instead such individuals may compete for moralistic status and may, therefore, use the sanctioned status signals of their church (modesty, moral displays, charity) to compete. We urge caution, however, as the cell size for non-religious individuals in the attractive condition was seven and the relationship between belonging to a religious tradition and being spiritual is not simple. In any case, our findings suggest this might be a fruitful avenue for future research.

Taken together, these results suggest that both men and women use mates (or potential mates) as social signals and that they are aware of the effects of such signals on others’ evaluations of them. More importantly, these results help explain human mate choice and dating behavior in real world social networks. These are often suffused with social information, and mating decisions in them are often carefully calculated because they have broad social ramifications [40]. A man who flaunts the head cheerleader is sending powerful information to others about his status and mate value and this information may influence the way others perceive and react toward him. Put more generally, individuals do not simply use manifested cues (visible phenotypes) in isolation to make optimal mating decisions; they also use social information–reputations and the suspected social ramifications of their decisions–to guide their choices. This applies to a workplace, a college, or a town, as much as to a high school.

Although we focused on nonindependent mate choice in this article, our extension of signaling theory may be applicable to many other facets of human social interaction, such as same-sex friendships and group affiliation. Most broadly stated, what we have proposed is that signaling theory can be extended to understand better many aspects of human relationships. Researchers have pointed out, for example, that status spreads through association such that mere association with high status individuals tends to increase a person’s own status [41]. This status contagion may explain why many are willing to spend exorbitant amounts of time and money collecting “icons” such as autographs, coins, and other memorabilia from celebrities and other famous people [24]. From the perspective of signaling theory, this contagion is the result of the signaling function of social association. If a person is seen with the president of the United States, he or she is accorded status because that association is usually a reliable signal of status, prestige, or other desirable trait(s). Conversely, if a person is seen associating with low status individuals, he or she loses status because that is generally a reliable signal that he or she cannot (does not have the qualities to) associate with higher status people.

Future studies should address many questions and issues that stem from the current study and from our application of signaling theory to social relationships. For example, different experimental designs should be used, perhaps ones that actually have the participants flaunt or conceal. The use of ethnographic or social network analyses may help unpack the complicated nature of flaunting/concealing and mate choice in information rich social environments. Traits other than partner attractiveness should be manipulated, such as intelligence, social status, sense of humor, and talent. We suspect, for example, that women would be more likely than men to flaunt partners who possess cultural status [28]. Future studies should also assess the relationship between personality variables and flaunting by using valid personality measures. We are hopeful that such a framework will attract researchers from diverse disciplines (sociology, social psychology, evolutionary psychology, evolutionary biology, zoology, marketing) and that it will allow a more accurate understanding of the complicated and socially dynamic phenomenon of human relationships.

## Supporting Information

Figure S1 **Flaunting and concealing as a function of relationship status.**
(DOCX)

Figure S2 **Flaunting and concealing as a function of religious affiliation.**
(DOCX)

Table S1 **Means, standard deviations, and correlations among all major measured variables.**
(DOCX)

Supplementary Information S1 **Main supporting information packet with demographic and other data.**
(DOCX)

Supplementary Information S2 **Face ratings.**
(DOCX)

Supplementary Information S3 **Pamphlet 1 (given to experimental group).**
(DOCX)

Supplementary Information S4 **Pamphlet 2 (given to control group).**
(DOCX)
