# Supplementary material for: If You’ve Got It, Flaunt It: Humans Flaunt Attractive Partners to Enhance Their Status and Desirability
Source: PLoS One. 2013 Aug 15;8(8):e72000. doi: 10.1371/journal.pone.0072000 (PMC3744452; doi:10.1371/journal.pone.0072000)
Supplement: Figure S2 — Flaunting and concealing as a function of religious affiliation. (DOCX) [file pone.0072000.s002.docx]

Figure S2. Flaunting and concealing as a function of religious affiliation

Note. 0 represents no location preference and is considered neutral (4 on the 7-point location preference scale). We calculated preference for undergraduate or administrative location by subtracting the participants’ scores by 4. Bars represent standard errors.
