# Supplementary material for: If You’ve Got It, Flaunt It: Humans Flaunt Attractive Partners to Enhance Their Status and Desirability
Source: PLoS One. 2013 Aug 15;8(8):e72000. doi: 10.1371/journal.pone.0072000 (PMC3744452; doi:10.1371/journal.pone.0072000)
Supplement: Table S1 — Means, standard deviations, and correlations among all major measured variables. (DOCX) [file pone.0072000.s003.docx]

|  | **Means and SDs** | | | |  | | | | | | | | | | | | | |
| --- | --- | --- | --- | --- | --- | --- | --- | --- | --- | --- | --- | --- | --- | --- | --- | --- | --- | --- |
| **Variable** | **Men** | **SD** | **Women** | **SD** | **1** | **2** | **3** | **4** | **5** | **6** | **7** | **8** | **9** | **10** | **11** | **12** | **13** | **14** |
| 1. Anxious | 2.34 | 1.16 | 2.27 | 0.92 | -- | **31** | 1 | -16 | -4 | -14 | 7 | **24** | -4 | 3 | 9 | -14 | -11 | -4 |
| 2. Embarrassed | 1.84 | 1.09 | 2.17 | 0.98 | **41** | -- | **-25** | **-40** | -17 | -15 | -14 | 7 | -18 | -18 | 17 | **-28** | **-24** | **-32** |
| 3. Enthusiasm | 2.36 | 0.99 | 2.33 | 0.96 | 14 | -15 | -- | **43** | -4 | -6 | 33 | -5 | -14 | **27** | -4 | 9 | 9 | **28** |
| 4. Happy | 2.58 | 0.91 | 2.51 | 0.92 | -7 | 1 | **36** | -- | 10 | -7 | **57** | -6 | 0 | **32** | -11 | 14 | **35** | **49** |
| 5. Confidence | 2.81 | 0.89 | 2.61 | 0.82 | -15 | -13 | -11 | 0 | -- | -7 | -10 | -4 | 2 | -13 | 3 | -13 | -2 | -8 |
| 6. Competent | 2.78 | 0.83 | 2.71 | 3.62 | -5 | **-25** | 10 | 19 | 5 | -- | -11 | -1 | -5 | 5 | -2 | -11 | **-13** | -23 |
| 7. Status | 2.97 | 0.96 | 3.07 | 0.96 | -15 | **-45** | 16 | -7 | -6 | 3 | -- | -5 | 2 | **63** | -10 | **39** | **58** | **58** |
| 8.Competent | 3.19 | 1.05 | 2.85 | 1.02 | 4 | -14 | 6 | 2 | -6 | 16 | -14 | -- | 10 | -14 | -6 | -22 | -16 | -11 |
| 9. Intelligent | 3.75 | 0.87 | 3.11 | 0.89 | 1 | -24 | 18 | 5 | 0 | 8 | 18 | 5 | -- | -6 | -3 | -15 | 8 | 2 |
| 10. Confident | 3.03 | 1.08 | 2.84 | 1 | -22 | **-45** | 8 | 5 | 16 | -3 | **78** | -21 | 13 | -- | 7 | 22 | **31** | **33** |
|  |  |  |  |  |  |  |  |  |  |  |  |  |  |  |  |  |  |  |
| 11. Leader | 3.64 | 0.86 | 2.63 | 0.9 | 19 | **31** | 15 | 11 | 0 | -9 | -11 | 1 | -6 | -14 | -- | 1 | -10 | -21 |
| 12. Comfortable | 2.95 | 1.23 | 2.95 | 1.17 | -18 | **-42** | 22 | 12 | 9 | 8 | **44** | -13 | 17 | **41** | **-26** | -- | **61** | **50** |
|  |  |  |  |  |  |  |  |  |  |  |  |  |  |  |  |  |  |  |
| 13. Desirable | 3.3 | 1.23 | 3.23 | 1.11 | -22 | **-35** | 13 | 13 | 14 | -4 | **49** | -9 | 10 | **41** | -15 | **70** | -- | **58** |
| 14. Flaunt/Conceal | 0.04 | 1.81 | 0.13 | 1.57 | **-31** | **-48** | 20 | 14 | 13 | 17 | **74** | -23 | 21 | **71** | -14 | **64** | **67** | -- |

**Table S1**. Means, standard deviations, and correlations among variables.

Note: Correlations were multiplied by 100; Items in bold are significant at *p* < .05. The first six items (blue) refer to the first set of items in the pamphlet; the second seven items (purple) refer to the second set items in the pamphlet. In red are correlations for men; in black are correlations for women.
