# Supplementary material for: If You’ve Got It, Flaunt It: Humans Flaunt Attractive Partners to Enhance Their Status and Desirability
Source: PLoS One. 2013 Aug 15;8(8):e72000. doi: 10.1371/journal.pone.0072000 (PMC3744452; doi:10.1371/journal.pone.0072000)
Supplement: Supplementary Information S1 — Main supporting information packet with demographic and other data. (DOCX) [file pone.0072000.s004.docx]

**Supplementary Material for Winegard, B.M., Winegard B.M., & Geary, D.C, “If you’ve got it, flaunt it: Humans flaunt attractive partners for status and desirability”**

**CONTENT**
**METHOD**
Demographic information …………………………………………………….………..1
Moderator analyses .....………………………………………………………………....3
Personality variables ….....................................……………………………………..…3
Mediation analyses ……………………………………………………………………..4
Debriefing information......…………………………………………………………......9
Operationalizing our variables..………………………………………………………...9
References..........…………………………………………………………………..........9
Table S1.............................................................................................................................
Figure S1............................................................................................................................
Figure S2.............................................................................................................................
Photos and ratings..............................................................................................................
Pamphlet 1..........................................................................................................................
Pamphlet 2..........................................................................................................................

**Demographic information:**

***Men***

Relationship status. 21 (32.8%) in a relationship (mean relationship satisfaction = 4.23, *SD* = 1.26), 0 married.

Self-identified Ethnicity. White = 52 (81.3%), Black = 4 (6%), Latino = 1 (2%), Asian = 5 (8%), Other = 2 (3%).

Undergraduate Major**.** Sciences (e.g., Chemistry, Physics) = 13 (20.3%), Social sciences (e.g., Psychology, Sociology) = 29 (45.3%), Humanities (e.g., History, English, Philosophy) 6 (9.4%), Other (e.g., Nursing, Business) = 16 (25%).

Religious affiliation (note, all Christian denominations were combined into the category “Christian,”). Christian = 29 (45.3%), Other = 9 (14.1%), None = 6 (9.4%), No response = 20 (31.3%)

Political affiliation. Republican = 23 (35.9%), Democrat = 14 (21.9%), Independent = 11 (17.2%), No response = 16 (25%).

GPA. 2.70, *SD* = .733. Six no Responses.

Number of close friends. 7.94, *SD* = 4.42.

***Women***

Relationship status. 28 (37.3%) in a relationship (mean relationship satisfaction = 4.28, *SD* = 1.18), 0 married.

Self-identified Ethnicity**.** White = 56 (74.7%), Black = 8 (10.6%), Latina = 0 (0%), Asian = 7 (9.3%), Other = 4 (5.3%)

Undergraduate major. Sciences (e.g., Chemistry, Physics) = 7 (9.3%), Social sciences (e.g., Psychology, Sociology) = 31 (41.3%), Humanities (e.g., History, English, Philosophy) 26 (34.7%), Other (e.g., Nursing, Business) = 11 (14.7%).

Religious affiliation (note, all Christian denominations were combined into the category “Christian,”). Christian = 38 (50.7%), Other = 4 (5.3%), None = 6 (8%), No response = 27 (36%).

Political affiliation. Republican = 29 (38.7%), Democrat = 19 (25.3%), Independent = 13 (17.3%), No response = 14 (18.7%).

GPA. 3.14, *SD* = .514. Four no Responses.

Number of close friends. 5.05, *SD* = 3.47.

**Additional Moderator Analyses**

We used Modprobe, a moderation analysis program for SAS and SPSS (Hayes & Matthes, 2009), to assess whether any of the demographic and participant information variables moderated the relationship between condition and flaunting/concealing. The only significant moderator was religious affiliation, coded as religious or non-religious.

**Demographic Variables**

Moderator *R*^2^ Change *F* *p****Age***

  .0001      0.0180  .8934
***Relationship status***
   .0006      0.0828  .7740
***Relationship satisfaction***
       .0452      2.4194  .1268
***Ethnicity***

  .0001      0.0148  .9035
***Undergraduate major***

  .0089      1.3386  .2493
***Religious affiliation***
      .0772   8.0953  .0055
***Political affiliation***

  .0034      0.3985  .5292
***GPA***
   .0011      0.1476  .7015
***Number of close friends***       .0043      0.6314  .4282

**Personality Variables**

**Trait:     M    SD  α**
***Extraversion***                        4.64 1.27 .631
***Agreeableness***          4.87 1.14 .528
***Conscientiousness***          4.84 1.07 .643
***Emotional Stability***             4.54 1.06 .434
***Openness***                             4.92 1.02 .644

**Moderator Analysis with Personality**
Moderator *R*^2^ Change *F P****Extraversion***

  .0061     .9277 .3372
***Agreeableness***
 .0024     .3647  .5469
***Conscientiousness***
  .0012     .1784  .6734
***Emotional Stability***
   .0072     1.1182 .2922
***Openness***

  .0099     1.5057   .2219

Because the personality scale we used has relatively low alphas (Gosling, Rentfrow, & Swann, 2003; see text), we note that any results should be interpreted with caution. Further, we do not report more detailed analyses, because the alphas dropped to an unacceptable level (α < .50) when we attempted to explore the relation between personality and flaunting/concealing within sex or relationship status. The scale was included in our pamphlets to enhance the realism of our cover story. We had no a priori hypotheses about the effects of personality traits on flaunting/concealing behaviors. At this time, the relation between personality and tendency to flaunt attractive partners or conceal unattractive ones cannot be determined. Future studies, using longer more reliable measures of personality will be needed to assess the potential relation between any of the Big Five traits and flaunting/concealing.

**Additional Mediation Analyses**

Below we present results of mediation analyses separately for men and women, followed by analyses for participants who reported being in a romantic relationship and those who reported being single. .

None of the six questions about how participants anticipated they would feel (e.g., anxious, confident, etc.) mediated the flaunting/concealing results nor did the other five questions (e.g. being confident, being a leader, etc.) concerning how participants believed others would perceive them.

**Mediation Analysis for Men only (n = 64)**

***IV to Mediators (a paths)***
            Coeff        *se         t         p*
Expected status        .3988     .1387    2.8755      .0055
Expected desirability     .3292     .1848    1.7817     .0797

***Direct Effects of Mediators on DV (b paths)***
            Coeff        *se         t          p*
Expected status      .9561     .1618    5.9087     .0000
Expected desirability     .5802     .1215    4.7762     .0000

***Total Effect of IV on DV (c path)***
           Coeff        *se         t          p*
Partner Status   . .8465     .2569    3.2956     .0016

***Direct Effect of IV on DV (c-prime path)***
           Coeff        se         t          p
Partner Status      .2742     .1679    1.6331     .1077

***Model Summary for DV Model***
     R^2^       Adj R^2^      *F                 p*
    .6908     .6754      44.6858       .0000

***Indirect Effects of IV on DV through Proposed Mediators (ab paths)***
           Effect      *se         Z                p*
TOTAL         .5723     .2112    2.7102     .0067
Expected Status          .3813     .1459    2.6144     .0089
Expected Desirability       .1910     .1133    1.6861     .0918

**Mediation Analysis for Women only (n = 75)**

***IV to Mediators (a paths)*** *Coeff        se         t         p*Expected status        .3231     .1294    2.4979     .0147
Expected desirability     .2142     .1532    1.3979     .1664

***Direct Effects of Mediators on DV (b paths)*** *Coeff        se         t          p*Expected status      .5777     .1848    3.1261     .0000
Expected desirability     .4971     .1560    3.1864     .0000

***Total Effect of IV on DV (c path)*** *Coeff        se         t          p*Partner Status   . .5134     .2132    2.4078     .0186

***Direct Effect of IV on DV (c-prime path)*** *Coeff        se         t          p*Partner Status      .2202     .1757    1.2537     .2141

***Model Summary for DV Model*** *R^2^       Adj R^2^         F                p*
    .4365     .4127      18.3307       .0000

***Indirect Effects of IV on DV through Proposed Mediators (ab paths)*** *Effect      se         Z                p*TOTAL         .2931     .1411    2.0780     .0377
Expected status        .1867     .0945    1.9753     .0482
Expected desirability      .1065     .0824    1.2917     .1964

**Mediation Analysis: Single participants (n = 90)**

***IV to Mediators (a paths)*** *Coeff        se         t         p*Expected status        .2963     .1101    2.6904     .0085
Expected desirability     .2439     .1451    1.6805     .0964

***Direct Effects of Mediators on DV (b paths)*** *Coeff        se         t          p*Expected status      .7288     .1632    4.4648     .0000
Expected desirability     .5608     .1239    4.5278     .0000

***Total Effect of IV on DV (c path)*** *Coeff        se         t          p*Partner Status   . .6143     .2021    3.0398     .0031

***Direct Effect of IV on DV (c-prime path)*** *Coeff        se         t          p*Partner Status      .2616     .1549    1.6892     .0948

***Model Summary for DV Model*** *R^2^     Adj R^2^         F                p*    .5212     .5045      31.2094       .0000

***Indirect Effects of IV on DV through Proposed Mediators (ab paths)*** *Effect      se         Z                p*TOTAL         .3527     .1443    2.4441     .0145
Expected status        .2159     .0929    2.3245     .0201
Expected desirability      .1368     .0862    1.5866     .1126

**Mediation Analysis: Participants in a relationship (n = 90).**

***IV to Mediators (a paths)*** *Coeff        se         t         p*Expected status        .4412     .1695    2.6028     .0123
Expected desirability     .2941     .2049    1.4358     .1577

***Direct Effects of Mediators on DV (b paths)*** *Coeff        se         t          p*Expected status      .7742     .2055    3.7679     .0005
Expected desirability     .4966     .1700    2.9208     .0054

***Total Effect of IV on DV (c path)*** *Coeff        se         t          p*Partner Status   . .7257     .2827    2.5668     .0135

***Direct Effect of IV on DV (c-prime path)*** *Coeff        se         t          p*Partner Status      .2381     .2113    1.1266     .2659

***Model Summary for DV Model*** *R^2^      Adj R^2^         F                p*    .5899     .5626      21.5805       .0000

***Indirect Effects of IV on DV through Proposed Mediators (ab paths)*** *Effect      se         Z                p*TOTAL         .4876     .2167    2.2504     .0244
Expected status        .3416     .1567    2.1792     .0293
Expected desirability      .1461     .1117    1.3076     .1910

**Debriefing**

Due to concerns that subjects may have suspicions about our cover story as well as ethical concerns about the manipulation, all participants were orally debriefed and probed to ascertain their state of mind during the experiment. Participants were told the true nature of the experiment and were provided with an explanation for the experimental design. They were encouraged to ask questions and voice concerns. None of the participants voiced serious concerns nor did any express that they found the instructions strange, although five men expressed disappointment that they would not actually collect survey data with their (attractive) “partner,” and three women expressed relief that they would not have to collect survey data with their (unattractive) “partner.” Two men also stated that they suspected that they were being deceived but reported that it did not affect their answers (“I was hedging my bets!” to quote one participant). We did not exclude their data. The oral debriefing concluded when all participants reported that their concerns and questions had been addressed.

**References**

Hayes, A. F., & Matthes, J. (2009). Computational procedures for probing interactions in OLS and logistic regression: SPSS and SAS implementations.  *Behavior Research Methods*, *41*, 924-936**.**

v
