# Supplementary material for: If You’ve Got It, Flaunt It: Humans Flaunt Attractive Partners to Enhance Their Status and Desirability
Source: PLoS One. 2013 Aug 15;8(8):e72000. doi: 10.1371/journal.pone.0072000 (PMC3744452; doi:10.1371/journal.pone.0072000)
Supplement: Supplementary Information S2 — Face ratings. (DOCX) [file pone.0072000.s005.docx]

**Men’s pictures** – **Attractive Condition** ratings (1-9) and standard deviations below picture.

Image 1) M = 7.73, SD = 1.16 Image 2) M = 7.47, SD = 0.92

Image 3) M = 7.47, SD = 1.13

**Men’s pictures** – **Unattractive Condition** ratings (1-9) and standard deviations below picture.

Image 1) M = 1.33, SD = 0.62 Image 2) M = 1.47, SD = 0.64

Image 3) M = 1.53, SD = 0.74

Women’s pictures – **Attractive Condition** ratings (1-9) and standard deviations.

Image 1) M = 8.31, SD = 0.87 Image 2) M = 7.38, SD = 1.02

Image 3) M = 8.38, SD = 0.80

Women’s pictures – **Unattractive Condition** ratings (1-9) and standard deviations.

Image 1) M = 1.50, SD = 0.63 Image 2) M = 1.94, SD = 1.12

Image 3) M = 1.56, SD = 0.72
