# Supplementary material for: If You’ve Got It, Flaunt It: Humans Flaunt Attractive Partners to Enhance Their Status and Desirability
Source: PLoS One. 2013 Aug 15;8(8):e72000. doi: 10.1371/journal.pone.0072000 (PMC3744452; doi:10.1371/journal.pone.0072000)
Supplement: Supplementary Information S4 — Pamphlet 2 (given to control group). (DOCX) [file pone.0072000.s007.docx]

Supplemental Pamphlet 2

Welcome to the Survey Collection Study on Higher Education

Please Wait For Instructions

In this study, we are interested in determining whether or not response rates and answers are influenced by the traits of survey givers. Previous studies, for example, have shown that couples giving surveys provoke more compliance and more positive reactions than singles. Research has also shown that personality variables affect the responses of those taking the surveys. We want to test these findings outside of the laboratory. You will survey individuals about higher education.  
Today, you will fill out a few surveys and choose a location and time to collect data on higher education. Other participants will be paired with an opposite individual. This group will be compared with the group of individual data collectors (you) to see if response rates and questions differ between groups.

When you go out to collect data, you should keep certain things in mind:


1) You will want to act as nice as possible and respect the feelings of the individuals you are collecting data from.

2) Please do not hassle or verbally abuse any of the individuals who you attempt to collect data from. You are not to argue with the expressed opinions of any individuals. Your job is to neutrally collect data.

**Please wait for further instructions before turning the page.**

Please answer the following demographic variables relevant to survey collection. If you do not feel comfortable answering a question, you may choose to leave it blank

1 What is your age? ______

2 Please check a mark by your gender   M__   F___

3 Please write your ethnic identification  _______________

4 Please list how many close friends you have _________

5 Please indicate your major______________

6 Please list your GPA ________

7 What is your relationship status?    Married ___    Single ___ Have girl/boyfriend ___

8 How satisfied are you with your current relationship?
Please answer by circling a number on the five point scale below. 1 indicates extremely unsatisfied, 3 is neutral, and 5 indicates extremely satisfied

1        2          3          4           5

9 What is your religious denomination (write “none” if you are not religious)? _________

10 What political party do you generally vote for?  Please check the box next to your answer.

Republican []    Democrat []  Independent []


Here are a number of personality traits that may or may not apply to you.  Please write a number next to each statement to indicate the extent to whichyou agree or disagree with that statement. You should rate the extent to which the pair of traits applies to you, even if one characteristic applies more strongly than the other.           

1 = Disagree strongly
2 = Disagree moderately
3 = Disagree a little
4 = Neither agree nor disagree
5 = Agree a little
6 = Agree moderately
7 = Agree strongly

I see myself as:

1.    _____  Extraverted, enthusiastic.

2.    _____  Critical, quarrelsome.

3.    _____  Dependable, self-disciplined.

4.    _____  Anxious, easily upset.

5.    _____  Open to new experiences, complex.

6.    _____  Reserved, quiet.

7.    _____  Sympathetic, warm.

8.    _____  Disorganized, careless.

9.    _____  Calm, emotionally stable.

10.  _____  Conventional, uncreative.


**Please wait for further instructions!!!!**

Available Times to Collect Data for our experiment. If you will not be available for any of the listed days, you may be contacted with a different set of days in the future. Conversely, you may be assigned other research instead.

The times listed are for two subsequent weeks FOLLOWING this week.

**WEEK 1 WEEK 2**

M T W TH F                                  M T W TH F

1pm  ____   _____     NA      ____     NA                             NA    ____        NA     ___     ____
2pm    NA  _____    NA      ____   _____                           ____     NA     ____    ____    ____
3pm  _____   _____   NA      NA     _____                           ____     NA     ____    ____    ____
4pm    NA     NA      ____     NA     _____                           NA       NA      ____   ____    ____
5pm    NA     NA      ____    ____    _____                           ____     NA     ____   ____    ____

When collecting data, you will have the choice of two locations described below. Remember that you will collect data exclusively from the people in your area. So, if you are in the undergraduate location, you will collect data from undergraduates and if you are in the administrative location, you will collect data from administrators and workers (e.g. secretaries and custodians).

**Undergraduate location:** This will be a heavily populated area of the campus consisting almost exclusively of undergraduate individuals between 19-25 years old. Often, there are groups of individuals from sororities and fraternities in this location. Depending on the time of day that you collect data, there may be individuals from other campus groups (such as sports club members). Your job will be to collect data from 10 individuals in this location.

**Administrative location:** This will be a heavily populated area of the campus consisting almost exclusively of individuals in their 40s and 50s who work on the campus. Often, there are campus custodians, secretaries, and maintenance workers in this location. Depending on the time of day that you collect data, there may be a few professors from other departments (e.g., english, anthropology). Your job will be to collect data from 10 individuals in this location.

Please answer the question below honesty:
LOCATION PREFERENCE: Please MARK the box you prefer.

1) I very strongly prefer the administrative (e.g. custodians) location

2) I strongly prefer the administrative location

3) I very slightly prefer the administrative location

4) I have no preference

5) I very slightly prefer the undergraduate (e.g. female and male undergraduates) location

6) I strongly prefer the undergraduate location

7) I very strongly prefer the undergraduate location

**PLEASE WAIT FOR FURTHER INSTRUCTIONS!!!!**

**When collecting the data how do you anticipate you will feel?**

The following will be completed on a 4 point scale. Please circle the appropriate number.

1 = not at all  2  =slightly 3 =moderately   4 =extremely  

Anxious   1    2    3   4

Confident  1   2    3    4

Embarrassed  1   2    3    4

Enthusiastic   1   2   3   4

Happy   1  2   3   4

Competent   1  2   3   4

**Please go to the next page and finish the Survey questions.**

**When collecting the data how do you anticipate OTHER PEOPLE will view you?**

The following will be completed on a 5 point scale. Please circle the appropriate number.

1 = strongly disagree  2  =mildly disagree 3 =neither agree nor disagree   4 =mildly agree   5  =strongly agree

Other individuals will view me as:

Having status    1 2 3 4 5

Being competent 1 2 3 4 5

Being intelligent 1 2 3 4 5

Being confident             1 2 3 4 5        

Being a leader 1 2 3 4 5

Being socially comfortable 1 2 3 4 5

Being desirable 1 2 3 4 5


**Please Turn Pamphlet Over When You Have Finished!**
